# Brain-Computer Interface Priming for Cervical Transcutaneous Spinal Cord Stimulation Therapy: An Exploratory Case Study

**DOI:** 10.3389/fresc.2022.896766

**Published:** 2022-06-23

**Authors:** Ciarán McGeady, Aleksandra Vučković, Niraj Singh Tharu, Yong-Ping Zheng, Monzurul Alam

**Affiliations:** ^1^Centre for Rehabilitation Engineering, University of Glasgow, Glasgow, United Kingdom; ^2^Department of Biomedical Engineering, The Hong Kong Polytechnic University, Kowloon, Hong Kong SAR, China

**Keywords:** transcutaneous spinal cord stimulation, neuromodulation, rehabilitation, brain-computer interface, motor priming, spinal cord injury

## Abstract

Loss of arm and hand function is one of the most devastating consequences of cervical spinal cord injury (SCI). Although some residual functional neurons often pass the site of injury, recovery after SCI is extremely limited. Recent efforts have aimed to augment traditional rehabilitation by combining exercise-based training with techniques such as transcutaneous spinal cord stimulation (tSCS), and movement priming. Such methods have been linked with elevated corticospinal excitability, and enhanced neuroplastic effects following activity-based therapy. In the present study, we investigated the potential for facilitating tSCS-based exercise-training with brain-computer interface (BCI) motor priming. An individual with chronic AIS A cervical SCI with both sensory and motor complete tetraplegia participated in a two-phase cross-over intervention whereby they engaged in 15 sessions of intensive tSCS-mediated hand training for 1 h, 3 times/week, followed by a two week washout period, and a further 15 sessions of tSCS training with bimanual BCI motor priming preceding each session. We found using the Graded Redefined Assessment for Strength, Sensibility, and Prehension that the participant's arm and hand function improved considerably across each phase of the study: from 96/232 points at baseline, to 117/232 after tSCS training alone, and to 131/232 points after BCI priming with tSCS training, reflecting improved strength, sensation, and gross and fine motor skills. Improved motor scores and heightened perception to sharp sensations improved the neurological level of injury from C4 to C5 following training and improvements were generally maintained four weeks after the final training session. Although functional improvements were similar regardless of the presence of BCI priming, there was a moderate improvement of bilateral strength only when priming preceded tSCS training, perhaps suggesting a benefit of motor priming for tSCS training.

## 1. Introduction

One of the most devastating consequences of cervical spinal cord injury (SCI) is partial or complete loss of hand and arm function (1). Loss of upper-extremity function has a drastic impact on a person's level of independence and quality of life, and as such is often their greatest priority in terms of rehabilitation (1, 2). However, after an initial period of spontaneous recovery, a motor function plateau is reached and further meaningful recovery is rare (3). Yet it has been shown that even in cases of severe SCI, there are often spared functional neurons that pass the level of injury which may be utilized to promote additional recovery (4). Indeed, this fact underpins much of the current activity-based rehabilitation offered to people with SCI (2). Despite the best efforts of clinicians, physiotherapists, and patients themselves, however, functional outcomes following rehabilitation are modest at best. Efforts must be taken to enhance the effects of rehabilitation.

Transcutaneous spinal cord stimulation (tSCS) has recently been proposed as a method for augmenting traditional exercise-based therapies (5). This non-invasive technique involves delivering high frequency currents via surface electrodes at and around the spinal level of injury (5, 6). It has been suggested that electrical interaction with various spinal structures, including dorsal column fibres, the dorsal horn and posterior/dorsal roots, decreases the motor threshold, making voluntary motor control easier through residual descending pathways (7–9). Although few in number, studies investigating the effects of cervical tSCS on hand and arm function have reported promising results (10–14). Inanici et al. showed that six individuals with chronic cervical SCI improved upper-extremity function following tSCS-facilitated intensive functional task training, with improvements remaining 6 months after the end of training. Impressively, some participants were able to resume activities such as playing musical instruments (14).

A further strategy for facilitating exercise-based therapy concerns priming (15). Movement-based priming involves repetitive or continuous volitional motor engagement with the purpose of enhancing the effects of a subsequent therapy (15). Evidence suggests that mirror symmetric, bimanual motor priming can facilitate motor cortical excitability and increase the rate of motor learning in neurologically-intact and neurologically-impaired individuals (16–18). Improved bimanual coordination and control may also increase the likelihood of functional improvements being maintained outside of the clinic, owing to bimanual movements being critical for performing activities of daily life (19, 20). Owing to the multi-faceted nature of SCI pathology, it has been suggested that the future of SCI treatment will rely on combinational strategies (21). Hence, where tSCS has been used to modulate spinal excitability, movement priming or motor imagery priming may be used to target supraspinal (cortical) networks (8, 22, 23). It has been shown that motor cortical activity is often diminished in individuals with chronic SCI, owing to damaged motor pathways and non-use of affected limbs (24, 25), yet cortical activation is a critical determinant of muscle strength (26). Although there is no evidence to suggest that enhancing cortical activity alone would correlate with improved functional performance after SCI, it may offer a priming effect that could complement an efficacious rehabilitative intervention, such as tSCS-facilitated upper-extremity training.

In this article, we present a brain-computer interface (BCI) priming strategy, that translates sensorimotor rhythms recorded from the electroencephalogram (EEG), reflective of cortical activity during movement, into a control signal for an interactive priming paradigm (27). Benefits of BCI-based motor priming include enhanced participant engagement, the ability to upregulate sensorimotor cortical activity, and lastly it provides insight to the SCI participant's neurophysiological state, which may provide markers that reflect functional recovery (25, 28). We expected that a session of BCI motor priming before tSCS training could enhance the effects of tSCS training alone.

We tested this hypothesis by recruiting an individual with a complete cervical SCI, who acquired his injury 12 years prior to enrollment in this study, and was graded as American Spinal Injury Association Impairment Scale (AIS) category A. We first had the participant undertake a five-week program of intensive upper-limb training with multi-site tSCS delivered to the cervical region of the neck. After a two-week washout period, where no training was administered, the participant underwent a further five weeks of tSCS training with BCI motor priming preceding each session. We expected upper-limb motor function to improve across both phases of the study, in line with previous literature. However, we expected enhanced rates of recovery during the priming phase.

## 2. Methods

### 2.1. Participant Characteristics

A 40-year-old male with a chronic cervical SCI participated in this study. Prior to enrollment, his injury, which occurred 12 years before recruitment, was graded as ASI A, with a C4 neurological level of injury, according to the International Standards for Neurological Classification of SCI (ISNCSCI) (29).

This study was approved by the Human Subjects Ethics Sub-committee of the Hong Kong Polytechnic University (HSEARS20190121002; 9 Feb 2019) and the participant provided written informed consent.

### 2.2. Experimental Protocol

**Figure 1:**
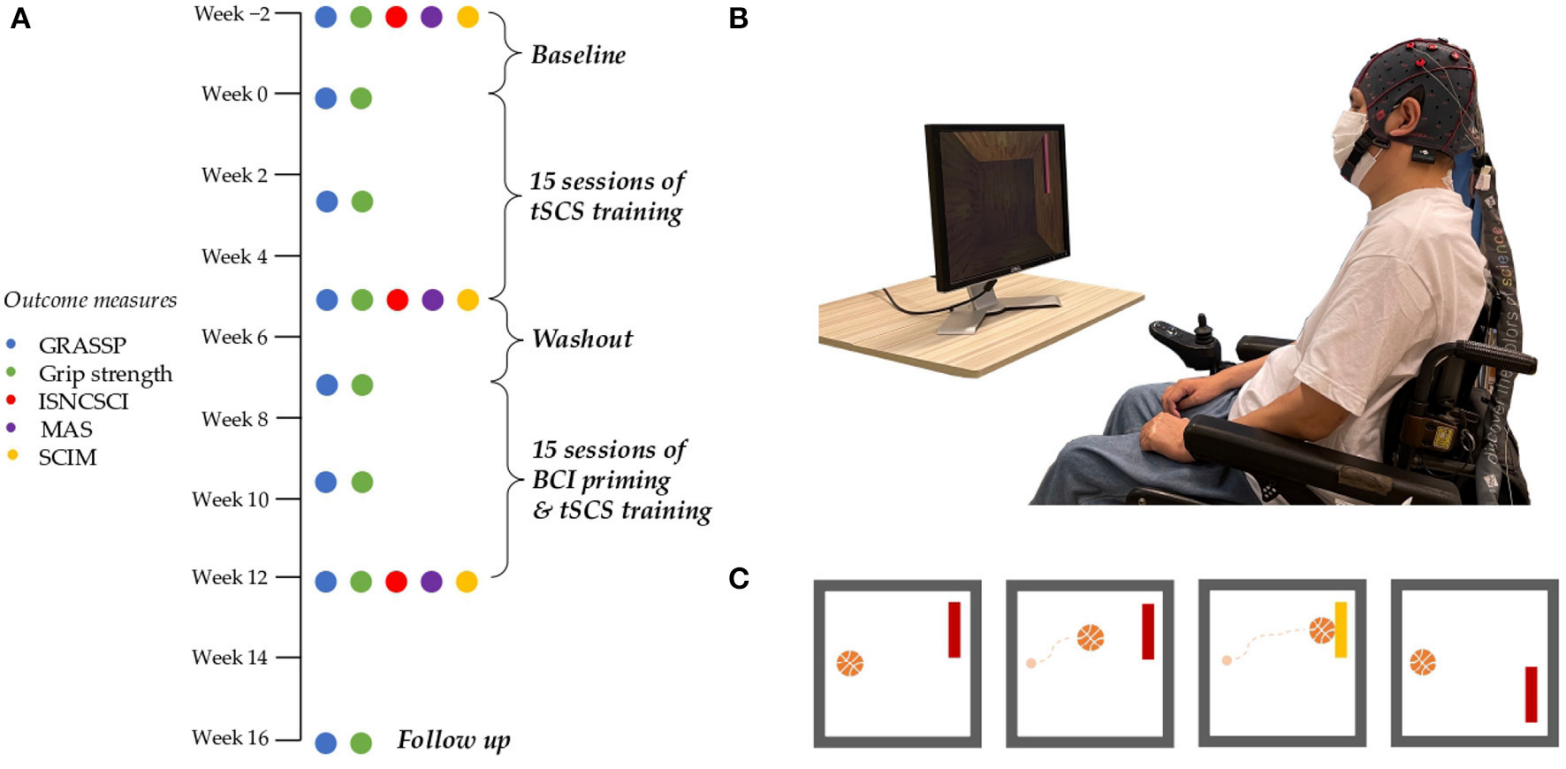
Study protocol and BCI motor priming paradigm. **(A)** The 18 week study protocol showing primary and secondary outcome measures. **(B)** BCI priming setup. The participant wore an EEG cap and sat opposite a computer screen. The participant provided written consent that his photograph be used in any publications. **(C)** A simplified version of the BCI motor priming paradigm as displayed on a computer screen and observed by the participant. The participant's objective was to attempt repetitive bimanual finger flexion/extension to guide a photo-realistic basketball to a target. The ball moved horizontally at a constant rate. Vertical displacement was influenced by the participant's EEG and his ability to engage with the priming task.

This study implemented a two-phase crossover design. After a two-week baseline period, the first phase involved five weeks of tSCS training three times per week, and a second phase introduced BCI motor priming before tSCS training for a further five weeks (Figure 1A) (10, 30). There was a two-week washout period between phases, and a follow-up assessment was conducted 4 weeks after the end of the second phase.

### 2.3. Hand and Arm Training

Hand and arm training consisted of repetitive uni- and bimanual exercises in conjunction with tSCS (31). A typical session focused on a number of grasp types, including palmar grasping, pinching, pinching with rotation, and finger isolation. Tasks included flipping playing cards, moving ping pong balls between containers, scooping rice with a spoon, and stacking blocks, among others. Tasks were adjusted relative to functional improvements to maintain a degree of difficulty. For example, ping pong balls were replaced with marbles and then by small beads as the study progressed. Hand training was performed continuously over the 60-min session with two brief pauses when the participant was given a break from tSCS.

### 2.4. Transcutaneous Spinal Cord Stimulation (tSCS)

A constant current stimulator (DS8R; Digitimer, Oxford, United Kingdom) delivered stimulation in bursts of ten 100 μs long biphasic rectangular pulses at a frequency of 30 Hz, reflecting recent clinical work (11, 13, 14, 32). Two round cathode electrodes (3.2 cm; Axelgaard Manufacturing Co, Fallbrook, CA, USA) were positioned at and below the level of injury between (i) C4 and C5, and (ii) C5 and C6 spinous processes. Cathodes were fastened to the skin with hypoallergenic tape to ensure a snug contact throughout the session. Inter-connected anode electrodes (8.9 × 5.0 cm; Axelgaard Manufacturing Co, Fallbrook, CA, USA) were placed symmetrically on the shoulders, above the acromion. In order to increase the likelihood of activating spinal structures, which lie below multiple layers of skin, fat, muscle, and vertebrae, stimulation intensity was set to highest tolerable degree (mean ± standard deviation; C4-C5: 49.0 ± 4.6 mA, C5-C6: 40.8 ± 5.1 mA) (8). Current intensity was determined at the beginning of each session by gradually increasing the current from zero mA in 2.5 mA increments. This continued until the participant verbally communicated that the stimulation was causing a painful sensation, as indicated by reference to the fifth increment (moderate–severe discomfort) of the Visual Analogue Scale for pain intensity (33). The participant reported habituation after prolonged stimulation, therefore stimulation intensity was re-evaluated after 10 min.

Stimulation was applied for a total of 60 min during each session. To avoid heating and skin irritation from prolonged high-intensity stimulation, there was a 2-min break every 20 min where the stimulator was switched off. Hemodynamic parameters (blood pressure and heart rate) were monitored during breaks to track any incidence of autonomic dysreflexia (34).

### 2.5. Brain-Computer Interface (BCI) Motor Priming

To motivate the participant to engage in motor priming, as well as record sensorimotor rhythms, we devised a game-like brain-computer interface priming paradigm based on the “BCI2000” platform (35). The participant positioned his wheelchair opposite a computer screen and was fitted with an EEG cap, as shown in Figure 1B. Conductive gel was injected into each electrode and signal quality was verified by visual inspection. Modulation of beta band power (14–25 Hz) from the participant's electroencephalogram (EEG) was used to guide a virtual basketball toward one of two targets. The participant underwent 300 repetitions of the priming task, divided into 10 runs, each separated by 10–60 s breaks to avoid fatigue. Each repetition, or “trial”, began with a photo-realistic basketball at the center-left of the computer screen, and a target either at the top-right or bottom-right (see Figure 1C). The ball moved horizontally at a fixed rate from left to right. The participant attempted mirror symmetric bimanual finger flexion and extension to push the ball to the upper target and relaxed for the ball to fall downwards. The participant was encouraged to imagine the sensation of clutching a real basketball as they performed the movement, in line with kinesthetic motor imagery protocols (36). Each trial lasted for 4 s and there was a 1.5–2.5 s inter-trial interval. Once priming was completed, the electrode gel was removed from the participant's hair, and the participant immediately proceeded to tSCS training.

In order to control the onscreen ball, EEG was recorded with a biosignal amplifier (g.USBamp; gtec, Schiedlberg, Austria) at a sampling rate of 256 Hz from ten active electrodes positioned at FC3, FC4, C1, C2, C3, C4, C5, C6, CP3, and CP4, according to the international 10-10 system (37). Electrode AFz was used as ground and the reference electrode was placed on the right earlobe. Through the BCI2000 platform, incoming EEG were spatially filtered with a small Laplacian filter to enhance the spatial resolution at electrodes C3 and C4, approximating the area above the sensorimotor cortices (35, 38). The spatially filtered data was transformed into the frequency domain using an autoregressive spectral estimation (39). The mid-beta frequency band (18–26 Hz) was found to be the most reactive band during movement and was used to influence the vertical trajectory of the ball. The sum of spectral power from electrode C3 and C4 was found every 50 ms from a 400 ms long window and vertical cursor control was determined by solving a linear equation. A detailed explanation of this procedure was described by Wolpaw and McFarland (35). A 5-min calibration session at the beginning of each session trained the program to classify between attempted movement and rest. The setup was identical to that of the above priming strategy. However, the ball only moved in the horizontal direction, with no vertical displacement.

The current setup required 30 min for BCI priming, including 10 min for setup and 5 min for equipment removal. The tSCS component required around 10 min to apply electrodes and establish stimulation parameters. Including breaks, a session of BCI priming with tSCS never exceeded 100 min.

### 2.6. Functional Outcomes

The Graded Redefined Assessment of Strength, Sensibility and Prehension (GRASSP) and grip strength were the primary measure of functional outcome (40). Grip strength was measured with the Vive Precision grip strength tester (Vive Health, Naples, FL, USA). GRASSP tested the strength of upper-limb muscles (Anterior deltoid, elbow flexors, elbow extensors, wrist extensors, extensor digitorum (DIII), opponens pollicis, flexor pollicis longus, finger flexors (DIII), finger abductors, first dorsal interossei), sensation on the dorsal and palmar sides of the hands, and fine and gross motor skills, quantified by scoring functional tasks (these included grasping and pouring water from a bottle, unscrewing the lids from jam jars, moving pegs between holes, inserting and rotating a key in a lock, inserting coins into a slot, screwing a nut onto a bolt).

Secondary outcome measures included the International Standards for Neurological Classification of Spinal Cord Injury (ISNCSCI) (29), and the Spinal Cord Independence Measure (SCIM) (41).

Further, the Modified Ashworth Scale (MAS) was used to quantify spasticity in the following upper-limb movements: shoulder abduction, elbow extension, elbow supination, wrist extension, and finger extension (42). The assessment was performed with the participant in the supine position and a trained physiotherapist graded each movement depending on the level of rigidity during flexion and extension. The minimum and maximum score for each unilateral movement was 0 (no spasticity) and 4 (velocity-dependent resistance to movement). A score of 1.5 was given when 1+ was selected [a detailed description of the MAS assessment was given by Charlambous et al. (42)]. The sum of scores from the left and right side were found for each movement.

All outcome measures were performed at the beginning and end of each intervention phase. Primary outcome measures were also performed in the middle of each five-week phase, and again at a 4-week follow-up session. Primary outcome measures were measured twice at baseline: once two weeks prior to the beginning of the first intervention phase, and once immediately before the first training session (refer to Figure 1A). Functional outcome measures were performed on different days from hand training sessions, and stimulation was not applied during any assessments.

### 2.7. Event-Related (De)Synchronization (ERD/ERS)

An offline analysis of the participant's EEG during BCI motor priming was performed to determine if sensorimotor cortical activity was modulated during and/or across sessions. EEG was first band-pass filtered from 1 to 40 Hz with a 3rd order Butterworth filter. Next, we calculated the power spectrum density during each trial, that is, from one to three seconds relative to the appearance of the ball (t=0 s). The pre-trial period (−1.5 to −0.5 s) was also found relative to the appearance of the ball. The mean power across the beta band (18–26 Hz) was subtracted from and divided by the mean of the resting state beta power to give the percentage ERD/ERS relative to pre-trial power.

## 3. Results

### 3.1. Graded Redefined Assessment for Strength, Sensibility, and Prehension (GRASSP)

**Figure 2:**
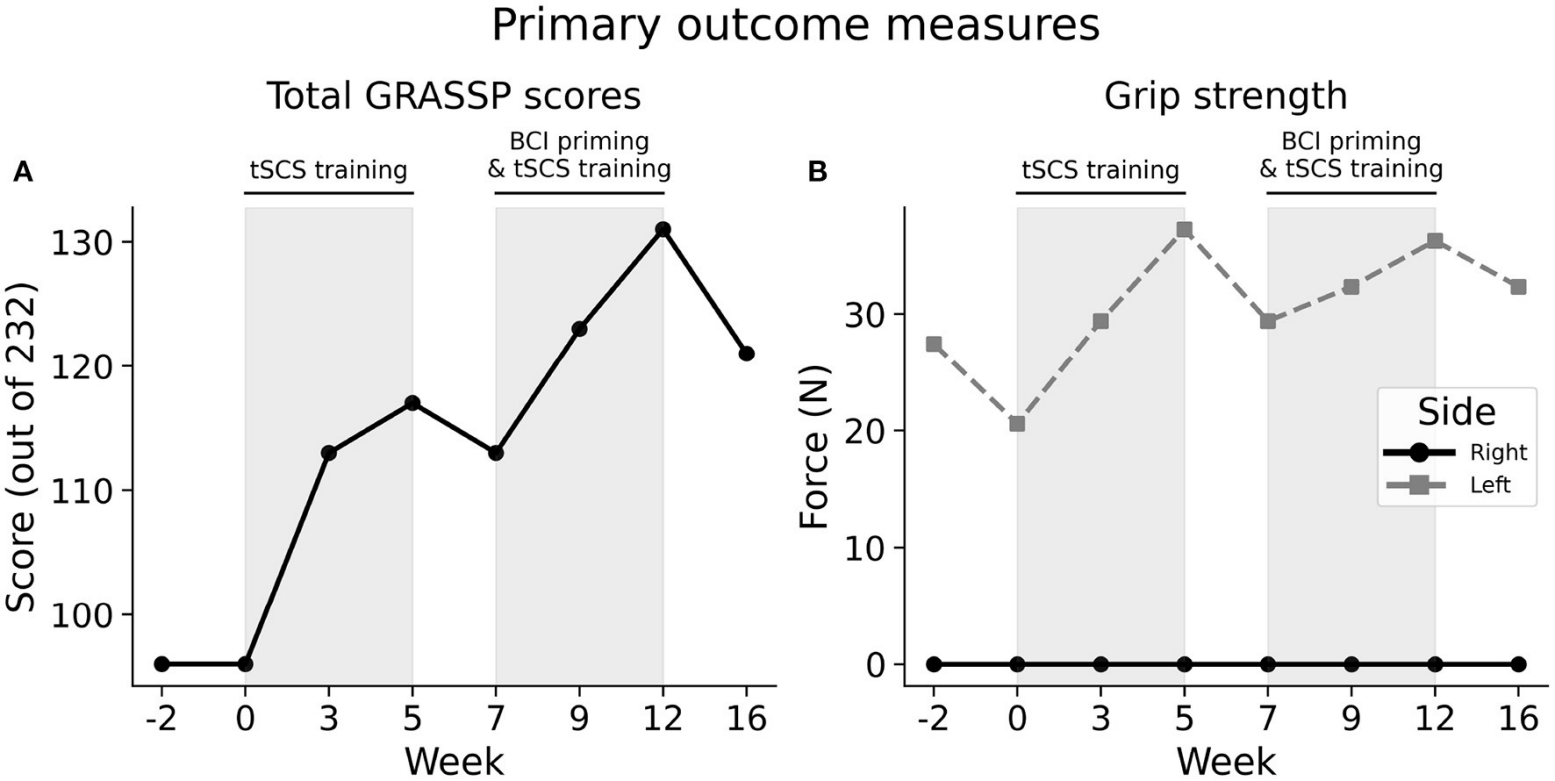
Upper-extremity primary outcome measures. **(A)** Score totals from the Graded Redefined Assessment for Strength, Sensibility, and Prehension (GRASSP) across the study. Shaded areas indicate the two therapeutic phases: “tSCS training” and “BCI priming & tSCS training”. **(B)** The participant's hand grip strength across study. The left and right hand is indicated with a gray dashed and solid black line, respectively.

At both baseline assessments, the participant scored a total of 96 out of 232 points in the Graded Redefined Assessment for Strength, Sensibility, and Prehension (GRASSP), as shown in Figure 2A. After five weeks of tSCS training this score increased by 21 points to 117/232, demonstrating improved upper-limb function. A two-week washout phase, where no training was administered, showed that functional gains were maintained, with only a slight, four-point drop in performance. The participant improved by a further 18 points to 131/232 following five weeks of BCI priming and tSCS training. A follow-up session 4 weeks after the final session showed that upper-limb functional improvements had generally been maintained, with a total GRASSP score of 121 points, a 26% increase in performance compared to baseline.

**Figure 3:**
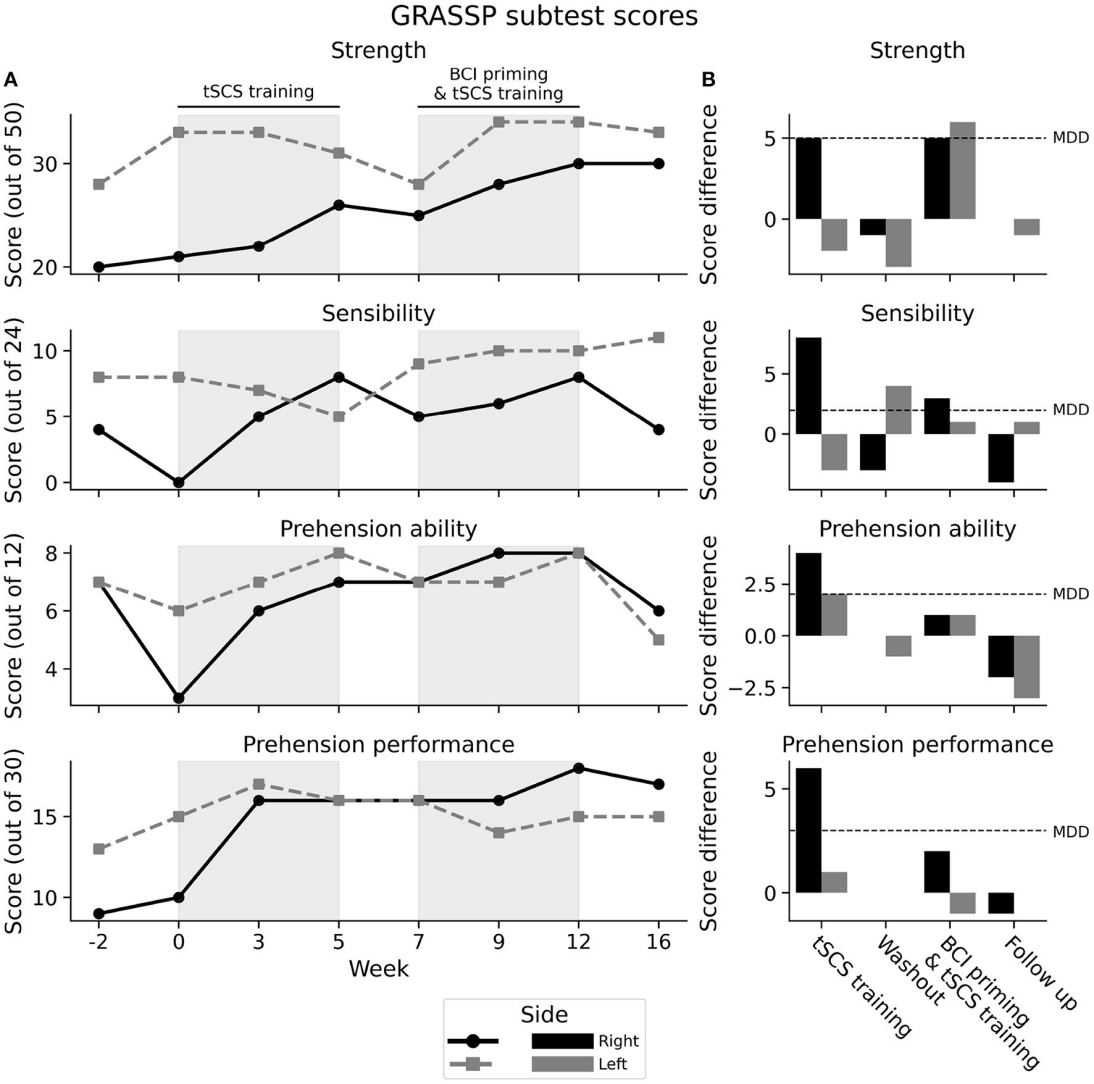
GRASSP subtest scores. **(A)** Subtest scores from the Graded Redefined Assessment for Strength, Sensibility, and Prehension (GRASSP) Strength across the study. Shaded areas indicate the two therapeutic phases: “tSCS training” and “BCI priming & tSCS training”. **(B)** Unilateral differences across each phase. Left and right side is indicated with gray and black, respectively. The minimally detectable difference (MDD) is the minimum score change required such that the difference cannot be attributed to measurement error (with 95% certainty). MDD is illustrated with a horizontal dashed line.

The right side was found to be more impaired that the left side at baseline in terms of strength, sensation of the hand, and ability to perform functional tasks. Improvements made during the first phase were generally attributable to the right side only, with strength, sensibility and prehension reaching to or exceeding the threshold for minimally detectable difference (MDD), Figure 3B. The MDD is the minimum amount of change in a participant's score that signifies that the change is not the result of measurement error (with 95% certainty) (43).

Strength in the left hand only improved when BCI priming preceded tSCS training, increasing by 6 points (28/50 to 34/50), above the MDD for unilateral strength (5 points). This strength gain was maintained 4 weeks after the final session. Improved score was attributable to contraction of the flexor pollicis longus, finger abductor, and first dorsal interossei, which had demonstrated no palpable contraction at the beginning of the second phase.

Sensibility, a measure of fingertip sensation, did not exceed MDD (more than 4 points) during either phase; I: +3.5 and II: +1, for dorsal sensibility, and −0.5 and +3 for palmar sensibility. There was, however, a 4.5-point increase in sensibility taking both phases into account, half a point above the MDD threshold.

The prehension subtest consisted of two domains: “ability”, a qualitative assessment of the participant's ability to position their hands in different grasping patterns—cylindrical grasp, lateral key pinch, and tip-to-tip pinch; and “performance”, measured by timing and scoring the participant as he performed functional tasks—such as entering a key into a lock, unscrewing lids from jam jars, and placing a nut on a bolt. Prehension ability improved by 4 points in the right hand and 2 points in the left hand following the first phase, meeting or exceeding the minimum detectable difference (2 points), see Figures 3A,B. The second phase did not improve this score beyond the MDD in either hand, and there was a drop beyond the MDD at the 1-month follow-up. Performance of the right hand showed great improvement after the first phase of tSCS training alone, increasing by 6 points (3 points beyond the MDD). This score improved by a further two points after the second phase of priming and tSCS training, one point short of the MDD. Interestingly, performance of the right had was maintained at the 1-month follow-up despite a drop in prehension ability. Performance of the left hand did not demonstrate the same improvements as the right hand, with only a one point increase after the first phase, and a one point decrease after the second phase, which was maintained by the 4-week follow-up.

### 3.2. Grip Strength

At baseline, the participant could produce 24.03 N of force with his left hand (Figure 2B). This increased to 37.27 N after the first phase of tSCS training, but decreased by 7.85 N when training was removed during the washout phase. His strength increased again following the second phase of tSCS training with BCI priming to 36.28 N. His left hand grip strength remained improved compared to baseline 4 weeks after the final training session at 32.36 N. The participant was unable to exert a detectable force on the grip strength meter with his right hand at any stage of the study.

### 3.3. International Standards for Neurological Classification of Spinal Cord Injury (ISNCSCI)

At baseline, upper-extremity motor scores measured during the ISNCSCI test showed greater impairment of the right side (13 points) compared to the left side (18 points; see Table 1), mirroring the GRASSP ‘strength” subtest. After 15 sessions of tSCS training, the right elbow extensors improved by one point, showing active movement against some resistance. After a further 15 sessions of tSCS with BCI priming, right finger flexors showed signs of contraction, contrasting with total paralysis at baseline and after tSCS training alone. Improvements in the left upper-extremity were not as consistent. Elbow extensors increased by a single point following tSCS training alone, which was maintained at the final assessment. However, wrist extensors dropped a single point following tSCS training with BCI priming. In summary, upper-extremity strength tended to increase on the more impaired side, during both phases of the intervention. The left side saw inconsistent changes of the upper-extremity motor score.

**Table 1:** ISNCSCI scores during baseline, after 5 weeks of “tSCS training”, and after 5 weeks of “priming & tSCS training”.

	**UEMS**		**LT**		**PP**		**NLI**	**ASI**	**Motor level**	
	**R**	**L**	**R**	**L**	**R**	**L**			**R**	**L**
Baseline	13	18	10	12	9	12	C4	A	C7	C7
tSCS training	**14**	**19**	9	11	**11**	**14**	C4	A	C7	**C8**
Priming + tSCS training	**15**	18	**15**	9	**15**	**14**	**C5**	A	C7	**C8**

*Measures include, upper-extremity motor scores (UEMS), light touch (LT) and pin prick (PP) sensory scores, neurological level of injury (NLI), American Spinal Injury Association Impairment Scale (AIS) category, and motor level. Scores from right and left side are indicated with R and L, respectively. Bold denotes an increase from baseline*.

The participant's perception of a pin prick generally improved following each session of the study, with more prominent changes on the more impaired side, that is, the right side. After a modest two-points increase after tSCS training (9–11 points), right-side pin prick perception increased by four points after tSCS training with BCI priming (11–15 points). On the left side, pin prick sensation improved from 12 to 14 points after the first phase of tSCS training and was maintained by the end of the second phase of BCI priming and tSCS training.

The participant's ability to perceive a light touch was again enhanced more on the right side during the therapy. After a single-point decrease in light touch sensation after tSCS training alone (from 10 to 9 points), the right side improved by 6 points (from 9 to 15 points) after BCI priming and tSCS training. The left side saw reduced levels of light touch sensation following both arms of the study, with a one point decrease after tSCS training (12 to 11 points), and a two point decrease following tSCS training with motor priming (11 to 9 points).

Taking both light touch and pin prick sensation together, the most caudal dermatome with intact sensation was C4 at baseline and C4 after tSCS training alone. After BCI priming and tSCS training, however, intact sensation was detected at C5. Taking both sensory and motor function into account, the participant's neurological level of injury shifted by one spinal level, from C4 to C5. Additionally, the most caudal myotome capable of active movement against gravity was C7 on both sides at baseline. After tSCS training, the motor score on the left side improved to C8. This was maintained for the rest of the study.

### 3.4. Modified Ashworth Scale (MAS)

Spasticity was generally reduced across the study, with improvements following both phases. Table 2 shows that shoulder abduction stayed at grade zero throughout the research period, indicating no spasticity, whereas wrist extension remained at grade 1, indicating minimal resistance to passive extension. During elbow supination, spasticity decreased by 0.5 points after tSCS training alone (3 to 2.5 points) and by 2.5 points after priming with tSCS training (2.5 to 0), a considerable improvement. Spasticity was recorded as zero during elbow extension following tSCS training alone, a 2.5 point reduction in spasticity from baseline, but was again detected following the priming phase. Compared to baseline, spasticity was elevated during finger extension following both phases: by 1.5 points following tSCS training alone, and by 0.5 points after a further phase of BCI priming and tSCS training. In sum, spasticity decreased equally following each phase of the study.

**Table 2:** Modified Ashworth Scale.

	**Shoulder**		**Elbow**		**Elbow**		**Wrist**		**Finger**		**Total**	
	**abduction**		**extension**		**supination**		**extension**		**extension**		
	**R**	**L**	**R**	**L**	**R**	**L**	**R**	**L**	**R**	**L**	**R**	**L**
Baseline	0	0	1	1.5	1.5	1.5	0	1	1	1	3.5	5
tSCS training	0	0	**0**	**0**	**1**	1.5	0	1	1.5	2	**2.5**	**4.5**
Priming + tSCS training	0	0	1	**1**	**0**	**0**	0	1	1	1.5	**2**	**3.5**

*Scores are given in terms of right and left side, indicated by R and L, respectively. Bold text indicates an improvement from baseline*.

### 3.5. Spinal Cord Independence Measure (SCIM)

According to the SCIM questionnaire (Table 3), the participant reported the same level of independence in self-care, respiration and sphincter management, and mobility at each phase of the study, suggesting that the functional improvements detected by the GRASSP did not translate to activities of daily life. An improvement in ability to move in bed was reported after the second phase of the study.

**Table 3:** Spinal cord independence measure (SCIM).

	**Self care (20)**	**Respiration and sphincter management (40)**	**Mobility (40)**	**Total (100)**
Baseline	6	15	6	27
tSCS training	6	15	6	27
Priming + tSCS training	6	15	**8**	**29**

*Bold text indicates an improvement from baseline*.

### 3.6. BCI Motor Priming

**Figure 4:**
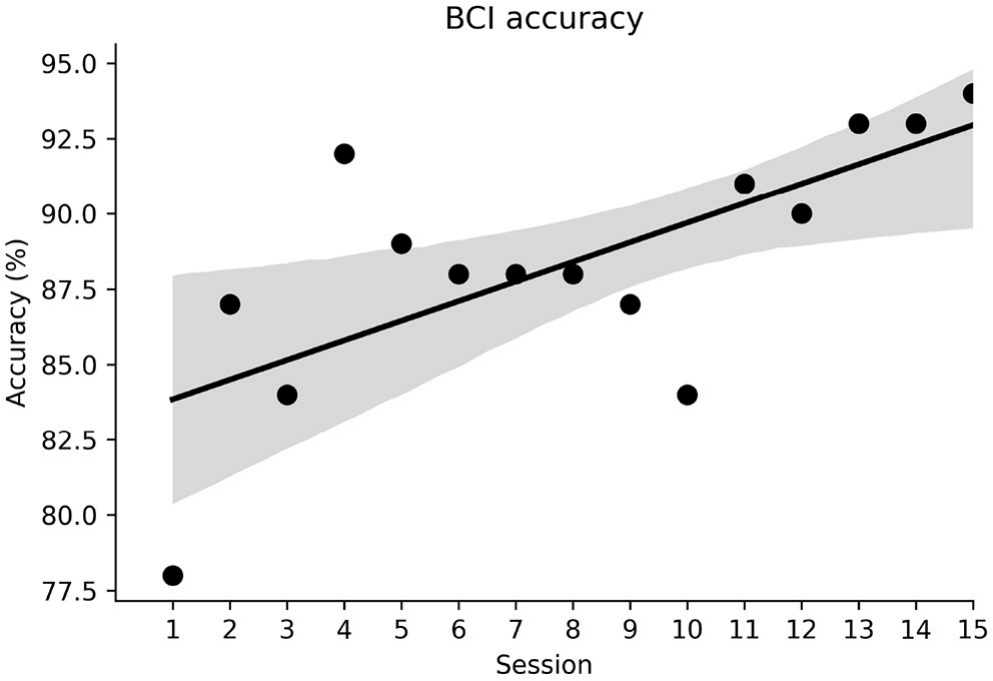
BCI classification accuracy across sessions. The shaded area represents the 95% confidence interval.

The accuracy of the BCI motor priming paradigm was defined as the percentage of successful target hits compared to the total number of trials. Figure 4 illustrates that the participant was able to modulate his sensorimotor rhythms efficiently across all priming sessions, with accuracies well above chance level (56%) (44). He successfully guided the ball to the correct target in 78% of trials during the first session and increased his accuracy to around 95% during the final sessions.

**Figure 5:**
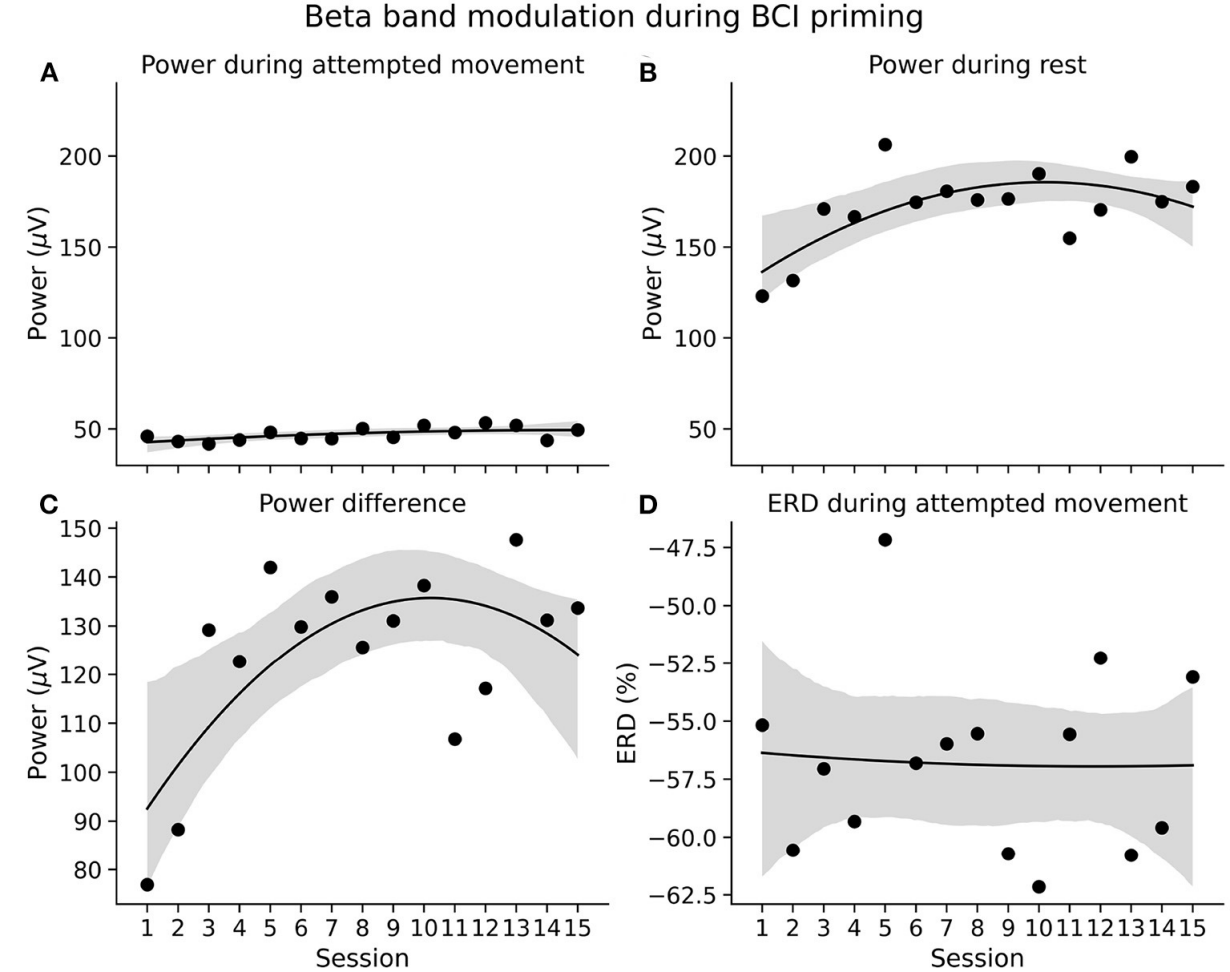
EEG characteristics during BCI motor priming. **(A,B)** Average beta band power during BCI priming conditions—attempted movement and rest, respectively—across sessions. **(C)** Difference in beta band power between priming conditions. **(D)** Beta band event-related desynchronization (ERD) during movement with respect to the pre-trial interval. The shaded areas represent the 95% confidence interval from a second order linear regression.

The improvements in classification accuracy were likely due to the participant becoming more adept at modulating his brain rhythms. Figures 5A,B show average beta band power during the two priming conditions—attempted movement and rest respectively—across the priming arm of the study. As expected, power during attempted movement is consistently lower than during rest. The difference in average power between conditions is shown in Figure 5C. Here it can be seen that the power difference widens over the first six sessions, before plateauing, indicating a learning process in the early sessions where the participant became increasingly able to induce distinct neural states. His ability to induce beta band event-related desynchonization (ERD) during attempted movement was consistent across the study at around −55%, as shown in Figure 5D. This suggests, therefore, that improved classification accuracies were likely associated with better control of the resting state.

### 3.7. Participant Compliance and Stimulation Intensity

**Figure 6:**
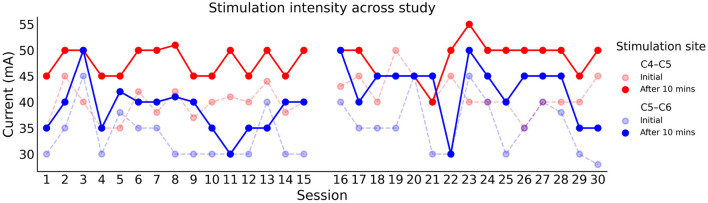
Stimulation intensity used in each training session across the study. The rostral (C4–C5) and caudal (C5–C6) electrodes are represented with red and blue respectively. The faded traces indicate the initial current intensity before habitation.

Stimulation intensity was determined at the beginning of each session by slowly increasing the current until the participant's maximum tolerance was reached. After 10 min of continuous stimulation, the participant was asked if he could tolerate a stronger intensity. As Figure 6 illustrates, the participant's maximum tolerance increased by around 10 mA in the vast majority of sessions, in both the rostral (C4–C5) and caudal (C5–C6) electrode. It can also be noted that the maximum tolerable intensity was consistently higher in the rostral electrode.

His maximum tolerable intensity at both stimulation sites was consistent across both arms of the study, at around 47 and 40 mA for the rostral and caudal electrode. It is of interest to note that these stimulation intensities were within the range previously demonstrated with able-bodied individuals using their maximum tolerance (45, 46).

The participant was well able to tolerate the stimulation and it did not impede his ability to engage with the activity or in conversation. His heart rate and blood pressure were stable throughout and across sessions.

The participant often attributed the discomfort of high intensity stimulation to excessive contraction of neck and back muscles. Occasionally, the participant reported a tingling in the hip region, a sensation he commented he had not experienced since before his injury.

## 4. Discussion

In this case study we investigated whether brain-computer interface motor priming could enhance upper-extremity function following intensive transcutaneous spinal cord stimulation training. After 15 sessions of tSCS training alone, the participant showed improved unilateral strength, sensation, and gross and fine motor control. After a further phase of combined BCI priming and tSCS training the participant made strength improvements bilaterally. This result may support the notion that the presence of a priming component in rehabilitative therapy could enhance the effect of a subsequent intervention. However, inconsistency across outcome measures tempers this notion. It may be that improved scores in the second phase were a continuation of the progress made in the first phase through tSCS alone. Future work is required to draw firm conclusions on the potential of priming for tSCS training.

The BCI component of the study relied on the modulation of the participant's EEG to control a computer game through attempted bimanual movement. Despite individuals with chronic SCI tending to display diminished sensorimotor cortical activity due to de-efferenation following injury (24), the participant was able to consistently modulate their sensorimotor rhythms, resulting in accurate control of the BCI priming paradigm, with improved performance over the course of the study. Good BCI performance indicated good compliance to the priming modality.

This work joins the growing literature supporting the use of tSCS for promoting functional recovery following SCI. There are strong parallels with a case study by Zhang et al. which reported that an individual with a chronic SCI improved their total GRASSP score beyond the threshold for minimal detectable difference, with improvements being most prominent in the strength and prehension category. Moreover, improvements were maintained above baseline at a 1-month follow-up assessment (13). On the other hand, the current results contrast with the work by Zhang et al. in terms of hand grip strength, which showed only mild improvement in the left hand and no improvement whatsoever in the right hand. Further, Zhang et al. reported immediate functional improvements in grip strength and motor control following tSCS onset. The current study reported no such instantaneous improvement. Only after multiple sessions of tSCS training did the participant begin to show improvements in strength and finger dexterity. This is somewhat surprising given that the participant in the current study had better upper-limb function at baseline compared to the individual described by Zhang et al., implying a greater volume of intact descending neurons.

Another notable study concerning upper-extremity tSCS training was conducted by Inanici et al. (14). Here, GRASSP was used to track the functional recovery of six chronic cervical SCI participants as they engaged in intensive tSCS-based physical practice. The impairment category tended to be less severe compared to the current study, with participants varying from AIS category B to D. Functional improvements were similar to the current study in the more severely injured participants, implying that tSCS training may be less suitable for those classified as AIS A. In addition to steady functional improvements over the course of multiple training sessions, Inanici et al. also reported improvements immediately following tSCS onset. It may be the case that electrode configuration played a role. In the two aforementioned studies, inter-connected anode electrodes were positioned symmetrically over the illiac crest. Whereas, in the current study, the anode was placed symmetrically over the shoulders. Electrode position has been shown to have a significant impact on the extent to which spinal structures are recruited by single-pulse stimulation (7). However, its importance on sub-threshold therapeutic stimulation is unclear. It may also be the case that a lack of instantaneous response to tSCS is a product of SCI pathology and not an instrumentation problem. An investigation of the mechanisms behind instantaneous vs. delayed performance enhancement would greatly benefit the field.

Surprisingly, the participant reported greater tolerance to stimulation at the rostral electrode, at odds with what one may expect given that sensory impairment tends to increase caudally from the injury level. However, this appears to be in line with ISNCSCI sensory scores. The participant's ability to detect a pin prick was intact until the fourth cervical dermatome, followed by altered sensation at C5, followed by intact sensation again from the sixth to seventh cervical dermatome, displaying a non-linear reduction in sensation. The greater tolerance to stimulation at the rostral electrode may be due to its location over this area of altered sensation.

In addition to GRASSP, we included grip strength as a primary outcome measure. This was measured with a digital grip dynamometer. The left hand showed increased strength after both phases. However, improvement was less pronounced during the second phase, potentially implying that priming did not provide an enhanced effect, or perhaps had a detrimental effect on tSCS training outcomes. Of the two baseline assessments performed, this interpretation relies on the measurement taken immediately preceding the first phase. The initial baseline measurement, however, showed greater left hand strength, demonstrating inter-session variability. This may be due to a variety of participant-related factors, such as fatigue and mood. If both baseline grip strength measurements are considered, the strength improvements during each phase become similar, implying that priming neither enhanced nor inhibited tSCS training. Moreover, despite GRASSP showing improved right hand strength, the right hand displayed zero grip strength at the beginning and throughout the study. We expect, however, that this was partially attributable to insufficient instrument sensitivity, and that right hand grip strength did improve to an extent, as indicated by the participant's increasing ability to perform grasping tasks across the study. A more complete view of the participant's grip strength would have been possible with a more sensitive measure of grip strength, targeting both cylindrical and pinch grasping.

Spinal cord stimulation has been shown to be a viable option for attenuating spasticity following SCI (14, 47, 48). The current study supports this notion in that MAS sum scores improved after each intervention phase. On individual muscles, however, there were inhomogeneous changes in spasticity. For example, elbow extension improved after the first phase but returned to baseline levels after the second phase. Aside from this perhaps being an issue of inter-rater reliability, it may be that agonist/antagonist groups were being activated in an inverse pattern due to multiple muscles being innervated by the C6/C7 myotome, lending a degree of variability to measures of spasticity. In the future, other measures should complement MAS, such as EMG-based evaluation of tonic stretch reflexes.

Safety is of prime concern during research with SCI participants, especially studies which administer noxious stimuli such as electrical stimulation. This is due to the potential for triggering autonomic dysreflexia, a life-threatening condition prevalent among people with cervical SCI (34). In the current study, we monitored the participant for signs of autonomic dysreflexia by tracking hemodynamic parameters, looking for signs of sudden facial flushing, or headaches, and by taking short breaks from tSCS. We found no adverse effects to stimulation. Occasionally, when the electrodes were removed at the end of a session, mild redness of the skin was observed. The skin in this area was not painful to the touch, and would fade within minutes–hours, in line with previous reports (49). Overall, the participant tolerated the multi-site stimulation well and did not report pain or annoyance. Paraesthesia in the arms and fingers was often reported by the participant. This was not unpleasant for the participant and was taken as a welcome marker of spinal cord stimulation (8).

In addition to safety, we were also concerned by the practical considerations involved in performing BCI and stimulation experiments in series, as both techniques required time to setup equipment while the participant was idle. This could fatigue the SCI participant and affect his willingness to engage with the intervention. The participant in the current study managed the setup time well and did not appear fatigued by the time required to setup equipment. Moving forwards, however, setup time can and should be optimized, for instance by reducing the number of EEG electrodes used for BCI priming. Moreover, we found that despite the setup time, the participant provided consistent effort for the duration of the priming sessions. This may be because the game-like nature encouraged him to actively participate, and the novelty of a BCI was interesting to him. This was desirable given that effort is a partial predictor of outcome in rehabilitation.

The use of priming for neurorehabilitation has gained momentum in recent years (15). Methods for priming the central nervous system have varied across studies, with strategies employing techniques such as motor imagery, action observation, and peripheral nerve stimulation (15, 18). In the present study, mirror symmetric movement priming was used to ready the upper-extremities for subsequent tSCS training. It has been previously demonstrated that 20 min of active-passive wrist flexion-extension can enhance corticospinal excitability in healthy participants (17). Here, it was hypothesized that a similar protocol could also elevate corticospinal network reactivity. However, quantifiable methods of excitability were not recorded. Future work should consider measuring corticospinal excitability–using techniques such as transcranial magnetic stimulation, or posterior root muscle reflexes–before and after BCI priming to verify a priming effect. Furthermore, efforts should be taken to understand the number of mirror symmetric repetitions required to ensure engagement of priming mechanisms.

Studies have shown that individuals with chronic SCI display reduced sensorimotor cortical activity during attempted movement of their impaired limbs (24, 25, 50). The participant in the current study had minimal motor and sensory ability of the distal upper-extremities. However, from the first session, his beta band showed strong levels of event-related desynchronization (ERD) during attempted bimanual finger flexion, implying strong activation of the sensorimotor representational areas responsible for eliciting finger flexion (51, 52). Indeed, it was expected that beta-band ERD would be relatively weaker at the beginning of the priming phase and show gradual strengthening over the course of the study. This would mirror similar work by Lopez-Larraz et al. which showed that ERD was significantly enhanced in a chronic C4 AIS A tetraplegic individual following four sessions of upper-alpha band neurofeedback training (25). Instead ERD was strong throughout the study and comparable to the activity of able-bodied individuals (46, 53). Perhaps this disparity is attributable to the participant in the current study having some residual control over his fingers, whereas the participant in the study by Lopez-Larazz et al. had no control below the elbow. Our rationale for priming with a brain-computer interface was to guide and quantify motor cortical activity during movement, such that enhanced activity would facilitate subsequent tSCS training. However, given that the individual in this current study exhibited maximal values of cortical activation at the beginning and throughout the study, it may be that supraspinal excitability was already at its greatest extent, making priming a superfluous addition. Potentially, priming of the nature described herein would benefit only those with impaired cortical activity, such as the variety described by Lopez-Larazz et al., and others (24, 25, 50).

Although improvements were relatively minor, demonstrated in part by minor changes noted in the Spinal Cord Independence Measure, the participant demonstrated improved control of his fingers, making some of the most demanding tasks, such as screwing a nut onto a bolt, possible. Improved total GRASSP scores were noted after every stretch of training. It is reasonable to assume that function would continue to improve given more sessions. Future work should consider training over a greater number of sessions to characterize the evolution of motor function. It would be valuable to determine whether BCI priming before tSCS training could reach a motor function plateau faster than tSCS training alone, or enhance the magnitude of recovery before plateau onset.

This current work sought to establish whether BCI motor priming could enhance the benefits of tSCS for SCI rehabilitation. To this end, our investigation assumed the efficacy of tSCS training *a priori*, given recent clinical studies (5, 14). For instance, Inanici et al. used a two-arm, cross-over study with two cross-over phases to demonstrate that tSCS-facilitated functional task training exceeded the therapeutic effects of functional task training alone. The current study did not follow such an approach, which may be considered a limitation, as it may be the case that similar functional improvements could have been achieved through hand training alone (2). To better understand how the combination of interventions may impact recovery a pilot study may be performed with multiple participants case matched into three groups: 1) training only, 2) tSCS training, and 3) BCI priming and tSCS training.

## 5. Conclusions

The aim of this work was to investigate whether BCI motor priming could improve the effect of transcutaneous spinal cord stimulation therapy in an individual with a cervical spinal cord injury. Following 15 sessions of intensive tSCS-facilitated hand training, the participant's upper-limb function improved in terms of strength, sensation, and ability to perform functional tasks. This was followed by a further 15 sessions of tSCS training, with the addition of BCI motor priming preceding each session. Improvements of a similar magnitude were recorded. However, no measure exceeded that which was achieved with tSCS training alone, with the exception of bilateral strength as measured with GRASSP. The power of this finding is diluted, however, given that strength, as measured during the ISNCSCI assessment, did not show the same pattern. It is likely that the GRASSP strength improvements in the second phase were a continuation from the first phase and would have occurred regardless of priming. The results of the current study do not eliminate the possibility that motor priming could impart meaningful efficacy upon tSCS training. Only through future work using a greater number of sessions, multiple cross-over phases, multiple case-matched participants, with comprehensive measures of corticospinal excitability, would allow for such a conclusion to be drawn. We hope that the current work is a meaningful step toward this robust study.

## Data Availability Statement

The raw data supporting the conclusions of this article will be made available by the authors, without undue reservation.

## Ethics Statement

The studies involving human participants were reviewed and approved by Human Subjects Ethics Sub-Committee of the Hong Kong Polytechnic University (HSEARS20190121002; 9 Feb 2019). The patients/participants provided their written informed consent to participate in this study. Written informed consent was obtained from the individual(s) for the publication of any potentially identifiable images or data included in this article.

## Author Contributions

CM, AV, and MA: conceptualization and validation. CM and MA: methodology and funding acquisition. CM: software, formal analysis, data curation, visualization, and writing—original draft preparation. CM and NS: investigation. Y-PZ: resources. AV, NS, Y-PZ, and MA: writing—review and editing. MA: project administration. All authors have read and agreed to the published version of the manuscript.

## Funding

This work was supported by RCUK Ph.D. scholarship EP/N509668/1, the University of Glasgow Graduate School Mobility Scholarship, The Hong Kong Polytechnic University (UAKB), and the Telefield Charitable Fund (83D1).

## Conflict of Interest

The authors declare that the research was conducted in the absence of any commercial or financial relationships that could be construed as a potential conflict of interest.

## Publisher's Note

All claims expressed in this article are solely those of the authors and do not necessarily represent those of their affiliated organizations, or those of the publisher, the editors and the reviewers. Any product that may be evaluated in this article, or claim that may be made by its manufacturer, is not guaranteed or endorsed by the publisher.
